# Pilot Study of Telehealth Delivered Rehabilitative Exercise for Youth With Concussion: The Mobile Subthreshold Exercise Program (MSTEP)

**DOI:** 10.3389/fped.2021.645814

**Published:** 2021-05-28

**Authors:** Sara P. D. Chrisman, Jason A. Mendoza, Chuan Zhou, Tonya M. Palermo, Tierra Gogue-Garcia, Kathleen F. Janz, Frederick P. Rivara

**Affiliations:** ^1^Center for Child Health, Behavior and Development, Seattle Children's Research Institute, Seattle, WA, United States; ^2^Department of Pediatrics, University of Washington, Seattle, WA, United States; ^3^Public Health Sciences Division, Fred Hutchinson Cancer Research Center, Seattle, WA, United States; ^4^Department of Anesthesiology and Pain Medicine, University of Washington, Seattle, WA, United States; ^5^Department of Health and Human Physiology, University of Iowa, Iowa City, IA, United States; ^6^Harborview Injury Prevention and Research Center, Seattle, WA, United States

**Keywords:** brain concussion, child, fear-avoidance, pain, exercise, physical activity, traumatic brain injury, sport

## Abstract

**Background:** Concussion is common, and up to 30% of youth develop persistent symptoms. Preliminary data suggests treatment with rehabilitative exercise is beneficial, but most programs require frequent in-person visits, which is challenging for youth in rural areas, and has been made more difficult for all youth during the COVID-19 pandemic. We have adapted an exercise intervention to be delivered via telehealth using Zoom and personal fitness devices, which could ensure access to this type of treatment.

**Objective:** The goal of this study was to assess feasibility and acceptability of a telehealth delivered exercise intervention for concussion, the Mobile Subthreshold Exercise Program (MSTEP), and collect pilot data regarding efficacy.

**Materials and Methods:** All youth received the 6-week MSTEP intervention which included wearing a Fitbit and setting exercise heartrate and duration goals weekly over Zoom with the research assistant. Youth completed standardized measures of concussive symptoms (Health Behavior Inventory, HBI), fear-avoidance (Fear of Pain Questionnaire, FOPQ) and health-related quality of life (Pediatric Quality of life Assessment, PedsQL), as well as a structured qualitative exit interview. We examined change in measures over time using mixed effects modeling, controlling for age, sex, prior concussion and duration of symptoms. We coded qualitative interviews using Thematic analysis.

**Results:** We recruited 19 subjects, 79% female with average age 14.3 (SD 2.2) and mean duration of symptoms 75.6 days (SD 33.7). Participants wore the Fitbit on 80% of days, and completed 94% of surveys and 96% of Zoom calls. Concussive symptoms (HBI) decreased significantly over the 6 week intervention (−10.6, 95%CI: −16.0 to −5.1) as did fear-avoidance (−21.6, 95%CI: −29.8 to −13.5). PedsQL improved significantly during the same time period (+15.1, 95%CI: 8.6–21.6). Approximately three-quarters (76%) of youth rated their care as “excellent.” Participants appreciated the structure of the guided exercise program and the support of the RA. They also enjoyed being able to track their progress with the Fitbit.

**Conclusion:** This study provides evidence for the feasibility and acceptability of a telehealth delivered rehabilitative exercise intervention for youth with concussion. Further research utilizing a randomized controlled trial is needed to assess efficacy.

**Clinical Trial Registration:** https://clinicaltrials.gov, identifier: NCT03691363. https://clinicaltrials.gov/ct2/show/NCT03691363

## Introduction

Estimates suggest up to 1.9 million youth sustain a concussion annually in the United States (1). While concussion normally resolves within days to weeks following injury, an estimated 15–30% of youth experience symptoms such as headache, fatigue, dizziness, and difficulty concentrating lasting more than 4 weeks (2, 3), currently referred to as Persistent Post-Concussive Symptoms (PPCS) (3). PPCS can confer marked functional impairment, interfering with academic performance and social interaction, and resulting in negative outcomes such as depression and school failure (4–6). Individuals who develop PPCS represent a small proportion of those injured, yet a disproportionate number of those requiring more intensive interventions and accruing medical expenses (6).

Research suggests a rehabilitative approach with sub-symptom threshold aerobic exercise may provide benefit for PPCS (7–16). Individuals with PPCS tend to have increased symptoms when engaging in physical activity (PA), and these symptoms can lead to avoidance of PA and subsequent disability (17). Studies have reported benefit of rehabilitative exercise for youth with concussion, thought due to retraining the autonomic nervous system, thereby facilitating more rapid recovery (8, 11, 13). Our prior study of an intervention using two in-person visits (Subthreshold Exercise Program, STEP) found benefit for aerobic exercise compared to an active control (stretching) (18). However, requiring in-person visits was challenging for youth who lived far from our urban location, and appeared to impede access.

Interventions delivered via telehealth improve access, generalizability and scalability of care (19). With technology-based interventions, treatment can be offered to youth in their homes, obviating the need to travel to distant clinical locations to receive subspecialty care. During the current COVID-19 pandemic there are additional advantages to delivering an intervention via telehealth, given that being seen in-person confers risk (20). As internet and mobile capacities have expanded, remotely administered telehealth interventions have proved efficacious for treating a broad array of medical issues, and encouraging health promotion (21). Telehealth treatment delivery has been particularly effective for increasing PA when paired with PA trackers (22, 23), and can improve adherence by utilizing more frequent touchpoints with participants (24).

Prior research on exercise as a treatment for PPCS has been grounded in the theory that physiologic change is responsible for treatment effects (8). We propose that positive outcomes associated with encouraging youth to exercise may also be mediated by psychologic change (18). In other words, youth with PPCS may have developed a fear-avoidance response to physical activity, similar to what has been described in youth with chronic pain (25–27). Other researchers have confirmed elevated levels of fear-avoidance in individuals with PPCS (28, 29), and our pilot study of an in-person delivered exercise program for concussion (the Subthreshold Exercise Program, or STEP) (18), demonstrated that fear-avoidance decreased in parallel with concussive symptoms (18). Interventions that encourage youth to exercise despite fears of exacerbating symptoms have been shown to be an effective approach to improving function in individuals with chronic pain (30).

Building from our in-person intervention (STEP), the goal of this study was to adapt the intervention to be delivered via telehealth (the Mobile Subthreshold Exercise Program, MSTEP) and to use mixed methods to assess the feasibility and acceptability of this approach. We also collected pilot data regarding treatment effects on primary outcomes (concussive symptoms and health-related quality of life) and impact on fear-avoidance.

## Materials and Methods

### Overview of Study

Methods were very similar to our previous study (18), but with the transition of all visits to telehealth. Subjects completed on-line surveys at baseline, 3 and 6 weeks via REDCap (31). The 6-week aerobic exercise program was delivered via weekly video conference calls with a research assistant (RA), advancing activity goals weekly. All subjects wore a Fitbit Charge 2 to allow them to track whether they were meeting activity goals. Youth and parents were provided incentives for participation, which were delivered after each task was completed.

### Sample

Youth were recruited during 2018–2019 through subspecialty concussion clinics (Sports Medicine and Rehabilitative Medicine) at Seattle Children's Hospital and the University of Washington by contacting families through a variety of means (texting, phone calls, and letters) to invite them to participate, as well as emailing providers in advance of a visit. Inclusion criteria included: (1) age 9–25 years old, (2) concussion occurring 1–9 months prior to the start of the study diagnosed by a clinician trained in concussion management consistent with the 2017 Berlin consensus definition of concussion (32), (3) PPCS as defined by the presence of at least three concussive symptoms rated at least 2 or greater on the Health and Behavior Inventory (HBI) (33), and a total score of 10 or greater. Exclusion criteria included: (1) parent and/or youth not fluent in English, (2) other injuries or medical conditions in addition to concussion that prompted a clinician to recommend against physical activity, (3) daily average of 30 min or greater of moderate to vigorous physical activity at time of enrollment, and (4) already completed a physical therapy intervention to increase aerobic exercise. Youth who chose to engage in the study continued to work with their concussion provider to receive usual care. The study was approved by the Institutional Review Board of Seattle Children's Research Institute. All youth and parents completed written informed consent. This study was registered at Clinicaltrials.gov #NCT03691363.

### Mobile Sub-threshold Exercise Program (MSTEP) Intervention

Subjects were asked to complete a home aerobic exercise program daily for 6 weeks. The exercise prescription included recommendations for frequency, duration and intensity in accordance with best practice (7). The initial goal was set at 10 min at a heart rate (HR) of 120 with an expectation that youth would attempt to exercise daily, but might miss 1–2 days per week. Individuals could choose the type of exercise they completed. If symptoms worsened during exercise, youth were instructed to take a break and decrease the heart rate goal utilized until they were able to tolerate 10 min of exercise. Goals were advanced weekly as tolerated to a maximum of 60 min of physical activity per day at a HR of 140. The HR of 140 was chosen as this approximates MVPA for youth (34, 35). The duration of 60 min/day was chosen as this is the US federally recommended level of MVPA for youth (36). Subjects were provided a Fitbit Charge 2 to monitor HR during their home exercise program and met with an RA weekly via video conference (Zoom) to discuss the progress of exercise that week, and advance goals for the next week. Zoom meetings took ~15 min and were scheduled at a time convenient for the participant.

### Assessments

#### Primary Outcome

The primary goal of the study was to assess feasibility and acceptability of the MSTEP intervention. Parents and youth completed an online survey using a standardized scale of patient satisfaction, the Satisfaction with Study questionnaire, consisting of 8 items such as “How would you rate the quality of care you have received?” and “Would you recommend this study to a friend.” They also completed structured qualitative interviews at the end of the study (via phone or video conference), in order to elucidate which parts of the study were most appealing and which could be improved. Interview questions were framed in an open-ended fashion and focused on participant experience with Fitbits, video conference calls and overall study procedures. Interviews were conducted by one of the RAs on the study using a standardized script, and were digitally recorded to allow for review and coding.

#### Secondary Outcomes

We collected pilot efficacy data regarding outcomes targeted by the intervention, including concussive symptoms, health-related quality of life, sleep, and symptoms of anxiety and depression. All scales were completed by youth via online self-report and included:

° Health and Behavior Inventory: The HBI is a component of the NIH Common Data Elements for research on concussion (37, 38), and is a 20-item instrument that measures the frequency of post-concussive symptoms on a four-point likert scale with higher scores indicating greater symptom severity. The scale yields scores in somatic and cognitive domains demonstrated by factor analysis to be robust across raters and time (Cronbach's alpha = 0.85–0.94) (33). This scale has demonstrated validity and reliability among adolescents and individuals with mild TBI (33, 39–42). Higher scores indicate worse concussion symptoms.
° Pediatric Quality of Life Inventory: The PedsQL is a 23-item 5-point questionnaire that assesses physical, emotional, social, and school functioning, including number of school days missed with established validity and reliability (43). Higher scores indicate better health-related quality of life.
° Fear of pain questionnaire, adapted for concussive symptoms: The FOPQ-C is a 24-item questionnaire, that has been shown to reliably and validly measure pain-related fear in youth (Cronbach's alpha 0.92) (44). Fear of pain is thought to arise from pain catastrophizing in the fear-avoidance model (25, 45). We adapted this measure to be specific to concussive symptoms, changing “pain” in each item to “concussive symptoms.” Higher scores indicate more fear and/or avoidance of concussive symptoms.
° Patient Health Questionnire-9: The PHQ-9 is a component of the NIH Common Data Elements for research on concussion. It is a 9-item instrument that measures depressive symptoms on a 4-point likert scale with higher scores indicating greater severity. This scale has demonstrated validity and reliability among adolescents and individuals with concussion (46–50).
° Generalized Anxiety Disorder Scale-7: The GAD-7 is a 7-item standardized anxiety measure that asks youth to rate how often they have been bothered by anxiety symptoms using a 0–3 scale (from “Not at all” to “Nearly every day”), with higher score indicating more severe anxiety. It has been shown to have good reliability, as well as criterion, construct, factorial, and procedural validity for assessing anxiety (51, 52).
° Adolescent Sleep Wake Scale-10 item: The ASWS is a 10-item scale regarding sleep quality that has been shown to have good internal consistency and construct validity (53). Higher scores indicate improved sleep quality.

### Covariates

Parents and youth completed additional surveys at the start of the study regarding demographic characteristics including: age, sex, race, ethnicity, parental education, and history of prior mental health diagnoses in youth and family members. Information was also collected regarding injury characteristics: date of injury (used to calculate duration of symptoms), mechanism of injury, primary symptoms experienced, and history of prior concussion.

#### Analysis

Data were examined for distribution and completeness. Data regarding satisfaction with the intervention were reported descriptively. Recordings of qualitative exit interviews were reviewed and coded iteratively using Thematic analysis to identify parts of the MSTEP intervention that were particularly liked or disliked (54). Changes in quantitative outcomes over time were examined using linear mixed effects regression models with time modeled as a discrete variable, while controlling for covariates of age, sex, duration of symptoms, and history of prior concussion. Subject-specific random intercept was included to account for clustering due to repeated measures within subjects. Fixed effect coefficients were tested using *F*-tests with Kenward–Roger methods for denominator degrees of freedom (55). All analyses were conducted using R statistical software (56).

## Results

### Sample

We approached 130 individuals, 78 were eligible, 16 declined, 16 were interested but did not follow through and 27 did not respond, leaving 19 who enrolled in the study. One individual withdrew from the study at 3 weeks due to increasing headaches. The sample was three-quarters female, average age 14.5 years (SD = 2.3 years), and majority white (63%, see Table 1). Duration of symptoms was about 2 months (average = 75.2 days, SD = 33.7) and all individuals reported headache, with difficulty concentrating and fatigue as the next most common symptoms.

**Table 1:** Demographics of youth participating in the Mobile Subthreshold Exercise Program (MSTEP) for concussion, Seattle, WA 2018–2019.

**Baseline characteristics (*N* = 19)**	***N***	**(%)**
**Age**		
10–13 y.o.	9	(47.37)
14–20 y.o.	10	(52.63)
**Female**	14	(73.68)
**BMI**^**a**^ **(kg/m**^**2**^**)**	Mean 23.17	SD (4.56)
**Race**		
White	12	(63.16)
African–American or Black	3	(15.79)
Asian	3	(15.79)
American Indian or Alaskan Native	1	(5.26)
Native Hawaiian or other Pacific Islander	0	–
Unknown	1	(5.26)
**Ethnicity**		
Hispanic	0	
Non-Hispanic	18	(94.74)
Unknown	1	(5.26)
**Education of consenting parent**		
HS or less	0	–
Some college	5	(26.32)
College degree	5	(26.32)
Masters or professional degree	8	(42.11)
Missing	1	(5.26)
**Education of other parent**		
HS or less	2	(10.53)
Some college	5	(26.32)
College degree	8	(42.11)
Masters or professional degree	3	(15.79)
**Family history (parent or sibling)**		
Headaches/ migraine	8	(42.11)
Neck/back pain	15	(78.95)
Joint pain	6	(31.58)
ADHD	5	(26.32)
Anxiety	8	(42.11)
Depression	4	(21.05)
Other mental health	2	(10.53)
Drug use/abuse	2	(10.53)
Alcoholism	1	(5.26)
Concussion or other brain injury	3	(15.79)
**Duration of symptoms**		
<60 days	7	(36.84)
61–95 days	7	(36.84)
96–150 days	5	(26.32)
**Mechanism of injury**		
MVC^b^	2	(10.53)
Fight/ hit by someone (i.e., assault)	0	–
Fell, not in sports	6	(31.58)
Sport or recreation related	11	(57.90)
**+LOC**^**c**^	4	(21.05)
**+Memory issues**	10	(52.63)
**Symptoms most problematic**		
a. Headache	19	(100)
b. Difficulty concentrating	14	(73.68)
c. Fatigue	12	(63.16)
d. Sensitivity to sound	12	(63.16)
e. Sensitivity to light	11	(57.89)
f. Memory issues	11	(57.89)
g. Dizziness	11	(57.89)
h. Balance problems	10	(52.63)
i. Irritability	9	(47.37)
j. Problems sleeping	8	(42.11)
k. Nausea	7	(36.84)
**Prior concussion**		
0	10	(52.63)
1	3	(15.79)
2	1	(5.26)
3+	5	(26.32)

a *BMI, Body mass index = weight in kg/ (height in m)^2^*.

b *MVC, Motor vehicle crash*.

c *LOC, Loss of consciousness*.

### Feasibility and Acceptability

Participants wore the Fitbit on 80% of days and completed 94% of surveys and 96% of Zoom calls. Both youth and parents expressed a high level of satisfaction with the study (see Table 2). More than three-quarters of youth and 69% of parents rated the study as “excellent,” and the remaining chose “good.” All parents and youth expressed that they would recommend the study to a friend. One parent and one youth expressed indifference or mild dissatisfaction on a few of the ratings.

**Table 2:** Satisfaction with Study ratings for youth and parents participating in the Mobile Subthreshold Exercise Program (MSTEP) for concussion, Seattle, WA 2018–2019.

	**Satisfaction with study (Youth** ***n*** **=** **17, Parent** ***n*** **=** **16)**	
	**Youth**	**Parent**
**How would you rate the quality of care you have received?**		
Excellent	13 (76%)	11 (69%)
Good	4 (24%)	5 (31%)
**Did you get the kind of care you wanted?**		
Yes, definitely	8 (47%)	12 (75%)
Yes, generally	9 (53%)	6 (25%)
**To what extent did these services meet your needs?**		
Almost all of my needs met	11 (65%)	11 (69%)
Most of my needs met	5 (29%)	4 (25%)
Only a few of my needs met	1 (6%)	1 (6%)
**Would you recommend these services to a friend?**		
Yes, definitely	11 (65%)	14 (88%)
Yes, I think so	6 (35%)	22 (13%)
**How satisfied are you with the amount of help you have received?**		
Very satisfied	12 (71%)	13 (81%)
Mostly satisfied	4 (24%)	2 (13%)
Indifferent or mildly dissatisfied	1 (6%)	1 (6%)
**Has the care you received helped you?**		
Yes, it has helped a great deal	11 (65%)	5 (31%)
Yes, it has helped	6 (35%)	9 (56%)
It didn't really help		1 (6%)
I do not wish to answer		1 (6%)
**In an overall general sense, how satisfied are you with the care you have received?**		
Very satisfied	14 (82%)	12 (75%)
Mostly satisfied	3 (18%)	4 (25%)
**Would you come back to this program?**		
Yes, definitely	11 (65%)	13 (81%)
Yes, I think so	5 (29%)	3 (19%)
I do not wish to answer	1 (6%)	

### Qualitative Interviews Regarding MSTEP Intervention

Exit interviews were completed by 79% of participants. Dominant themes suggested subjects overall had very positive experiences with the MSTEP intervention, particularly mentioning enjoying wearing the Fitbit, liking the structure of a gradual increase in exercise supported by an RA, and appreciating being able to get back to their sports and other activities (see Table 3). A fair number of youth mentioned that their symptoms had improved. A few youth had difficulties with syncing and charging the Fitbit, and a few discussed challenges in determining how many minutes they had achieved at their goal heart rate. Two youth mentioned symptoms worsening.

**Table 3:** Qualitative data from exit interviews with youth and parents participating in the Mobile Subthreshold Exercise Program (MSTEP) for concussion, Seattle WA 2018–2019.

**Qualitative themes from exit interviews** ** (Number in parentheses represents the number of interviews in which this theme was identified)**	**Representative quotes**
**Fitbit**	
1. Positive experiences with the Fitbit (*n* = 14)	• “It was really cool to see how many steps I could get in a day, and just like challenging myself with all of it.”
	• “Super easy to wear, just like a watch.”
	• “Easy to use, charged really fast.”
	• “Nice being able to see distance and pacing.”
2. Negative experiences with the Fitbit	
a. Need to sync manually (*n* = 5)	• “The automatic syncing didn't really seem to be working. …for the first couple weeks I always forgot that I had to open the app.”
	• “Syncing is poor, doesn't automatically sync.”
b. Hard to visualize heart rate (*n* = 5)	• “Liked being able to track heart rate, but hard to see it graphed in the app.”
	• “Doesn't display heart rate information in a way that is easy, hard to see how many minutes, have to kind of ‘eyeball it.”'
c. Trouble charging (*n* = 3)	• “Hung up by the way it had to charge, needed to have a certain direction.”
	• “Sometimes it would die in the middle of exercise.”
d. Difficulty measuring heart rate (*n* = 2)	• “Heart rate didn't seem very accurate, would say it was 100 when it was 150, but most of the time it was pretty close.”
	• “Had to make it tighter to get it to register the HR correctly.”
**Zoom**	
3. Positive experiences with Zoom (*n* = 7)	• “Liked everything about the weekly calls, it always connected and worked.”
	• “Calls were really easy to do, just click the link.”
4. Trouble connecting with Zoom (*n* = 4)	• “…The first two calls I was having a lot of problems figuring out how to work Zoom…after that I was able to get it down.”
	• “Originally getting the Zoom app to work was a little complicated, because I don't think it's something that anyone ever uses except for conference calls for work.”
**Overall**	
5. Liked having structure (*n* = 8)	• “It was helpful to have a plan instead of just trying to ‘wing it.”'
	• “Before…people told me to exercise, but I didn't know how much or where to start. This made it an easy process, like it was all laid out for me.”
	• “…Before entering into the study I was doing as much exercise as I could until I crashed, which wasn't the best way of doing it. The study kind of helped me get back to the basics and slow down so that I could go up and still feel good.”
6. Able to return to usual activities (*n* = 8)	• “Got me back to track practice, really accessible working around my schedule.”
	• “A couple of months ago I couldn't even go to school without headaches, now I can go to school, go to dance without symptoms.”
7. Symptom improvement *(n* = 6)	• “…It was a really fun experience, and it helped me get better in some ways…now I never really get blurred vision or dizziness anymore and headaches don't last as long and they're not as painful. “
	• “Helped me get rid of my concussion symptoms. For 2 months I wasn't getting any better, and then I started seeing results like 2 weeks in.”
8. Simplicity of the methods (*n* = 6)	• “Pretty much everything worked pretty well.”
	• “I feel like this is really nice and simple.”
	• “Appreciated how short the calls were (10–15 min).”
9. Appreciated RA support (*n* = 4)	• “I liked that I had someone to talk to.”
	• “Good that we could clarify stuff like if I had a question I could just ask.”
10. Advancing science (*n* = 3)	• “I liked the study a lot, I liked being a part of it and doing something that was good for other people, I liked how it all worked and it was just a fun experience.”
	• “Finding out more about concussions could definitely be a step in the right direction because I go to doctors all the time and they're like “Well-concussions are pretty unclear and we don't really know very much about them” and that's not very helpful.”
11. Increase in symptoms (*n* = 2)	• “Every time I do activity, the next day or two or three I feel really dizzy”
	• “Once we got to 150 beats, I started getting more headaches.”

### Outcome Data

**Figure 1:**
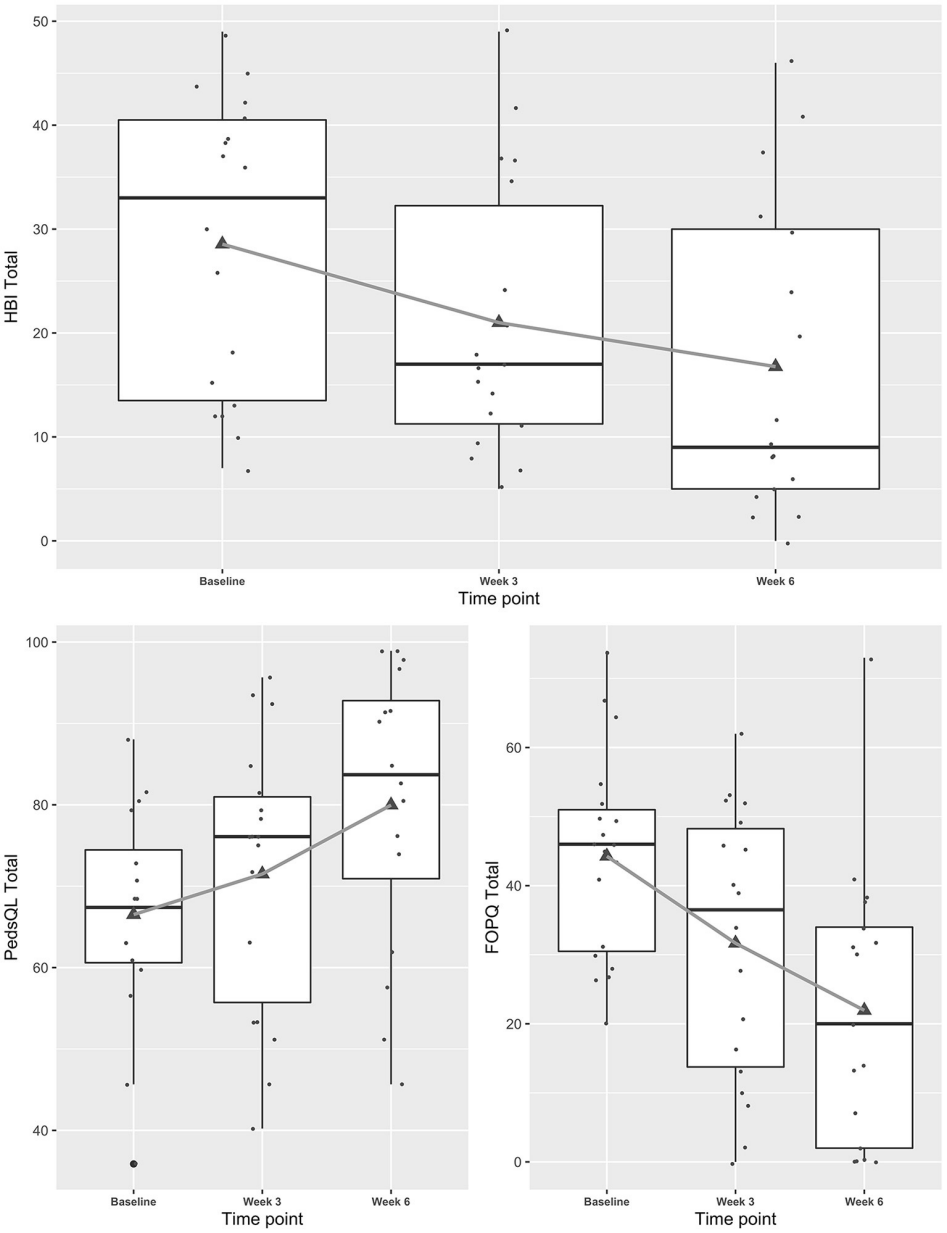
Trajectories of concussive symptoms, health-related quality of life and fear-avoidance for youth participating in a Mobile Subthreshold Exercise Program (MSTEP) for concussion, Seattle, WA 2018–2019.

**Figure 2:**
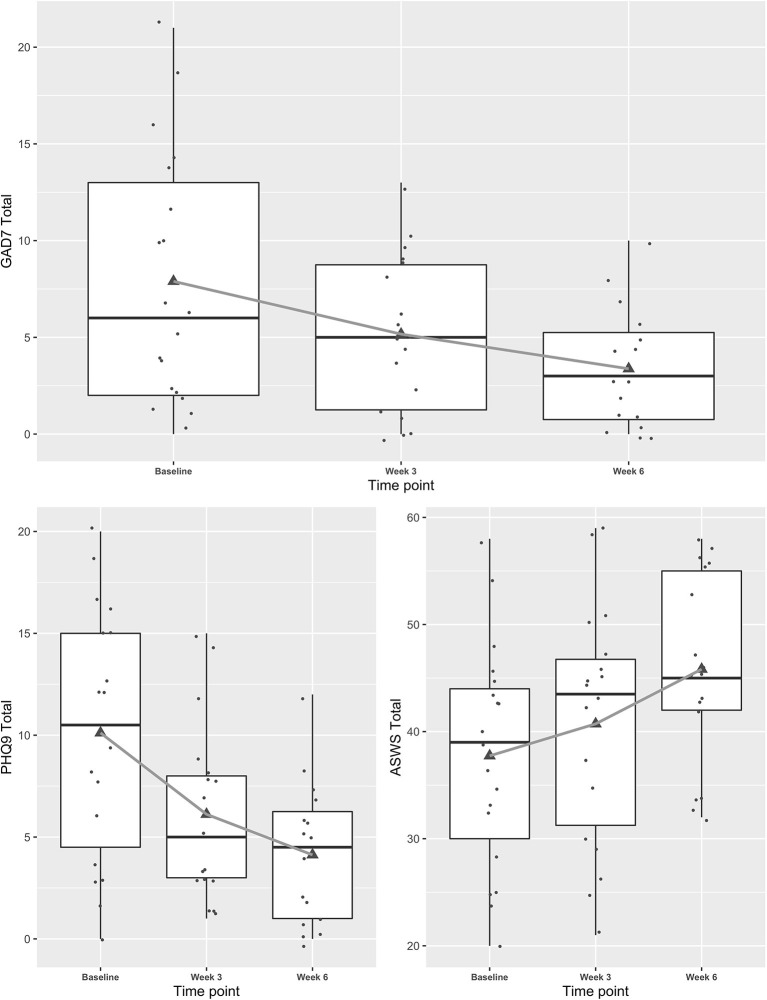
Trajectories of mental health symptoms and sleep for youth participating in a Mobile Subthreshold Exercise Program (MSTEP) for concussion, Seattle, WA 2018–2019.

Mixed effects regression models indicated concussive symptoms (HBI) improved significantly from baseline to weeks 3 and 6 while health-related quality of life (PedsQL) improved (Figure 1, and see Appendix for table). Fear-avoidance of concussive symptoms (FOPQ-C) declined significantly over the same time period (Figure 1), as did symptoms of anxiety (GAD7) and depression (PHQ9) (Figure 2). Sleep (ASWS) significantly improved at 6 weeks compared to baseline (Figure 2). Covariates were included in all models (age, sex, history of prior concussion, and duration of symptoms), but they did not affect model fit significantly (in the HBI model, likelihood ratio test comparing models with and without covariate adjustment had *p* = 0.22).

## Discussion

Research indicates that exercise is beneficial for treating youth with persistent post-concussive symptoms (PPCS), but prior studies have required multiple in-person visits (7–16). This is the first study to show that a rehabilitative exercise program for concussion (MSTEP) can be feasibly delivered via telehealth, a useful adaptation during a pandemic when in-person care is both challenging to access and higher risk (20). Youth and parents expressed high satisfaction with MSTEP, and would recommend this program to others. Youth in particular enjoyed the structured approach to returning to physical activity and the weekly video conference support from the RA. They also liked wearing the Fitbit, as it provided a means to assess whether they were meeting their activity goals. Technical difficulties were minimal and all were resolved during the study.

In order to adapt the study to be delivered via telehealth, we had to derive an alternate means for providing a tailored exercise program. Most in-person programs utilize a fitness test such as the Buffalo Concussion Treadmill Test to determine a target heart rate (57), which would be challenging to complete remotely as it requires a treadmill that can achieve a high level of grade. To replace this assessment, we designed a program that would target the approximate MVPA for a youth of the average age in the study (HR 140), and then asked participants to adjust the intensity based on their symptoms. Subjects tolerated this level of MVPA well and were comfortable making these adjustments. Only one participant ended up withdrawing from the study, supporting the acceptability of this approach.

Our preliminary analysis of quantitative outcomes suggested significant declines in concussive symptoms (HBI) during the 6-week intervention. Given that youth participants were enrolled following an injury, some level of improvement would be expected during the 6-week study, and the lack of a control group limits interpretation of declines in concussive symptoms. Future research with a randomized controlled trial is needed to ensure improvement in symptoms is not due to the passage of time. The MSTEP intervention effect (i.e., decline in concussive symptoms) was similar to an in-person exercise program (STEP) at 3 weeks, but slightly less strong at 6 weeks (18). We also noted improvements in depression, anxiety, sleep, and health-related quality of life, all of which paralleled declines in concussive symptoms. Future research will be needed to determine whether such improvements are due to resolution of concussive symptoms, or represent secondary endpoints. Fear-avoidance of concussive symptoms declined as it did in our previous pilot work (18), again suggesting that rehabilitative exercise addresses not only physiologic symptoms, but psychologic issues (such as fear of concussive symptoms) that may be responsible for symptom perpetuation.

This was a pilot study, and as such the sample size was small limiting generalizability. We note that the rate of recruitment appears low, which could introduce bias. However, in truth only 20% of youth declined participation. The remaining individuals either never responded to outreach (passive decline) or stopped responding. We suspect that many of these individuals recovered and therefore were no longer eligible, but this is difficult to verify. In any case, our recruitment numbers were comparable to a study by another group using in-person exercise to treat youth with PPCS (13). We also note that we did not have a concurrent control group for comparison and thus we cannot assess efficacy. Our next step is to conduct a larger randomized controlled trial of the MSTEP approach using an active control comparator (a stretching intervention) to assess the effect of the intervention on concussive symptoms and health-related quality of life, and examine potential mediators of the intervention effect such as fear-avoidance of concussive symptoms. We also plan to measure MVPA objectively using hip-mounted accelerometry, to assess whether increases in MVPA mediate recovery.

## Conclusions

A telehealth-delivered rehabilitative exercise program for youth with concussion (MSTEP) is both feasible and acceptable. A larger randomized controlled trial is needed to assess efficacy for this approach.

## Data Availability Statement

The raw data supporting the conclusions of this article will be made available by the authors, without undue reservation.

## Ethics Statement

The studies involving human participants were reviewed and approved by Seattle Children's Research Institute IRB. Written informed consent to participate in this study was provided by the participants' legal guardian/next of kin.

## Author Contributions

SC conceived and designed the study, obtained funding, coordinated data collection, oversaw analysis, drafted the manuscript, and submitted the final version. JM, TP, and FR contributed to the design of the study, supported data collection and analysis, provided critical revisions to the manuscript, and approved the final version. CZ completed the quantitative data analysis, provided critical revisions to the manuscript, and approved the final version. TG-G completed the data collection, cleaned and prepared data for final analysis provided critical revisions to the manuscript, and approved the final version. KJ provided insight into the design of the study (particularly the intervention methodology), supported data analysis, provided critical revisions to the manuscript, and approved the final version. All authors contributed to the article and approved the submitted version.

## Conflict of Interest

The authors declare that the research was conducted in the absence of any commercial or financial relationships that could be construed as a potential conflict of interest.

## Appendix

**Table A1:** Output from mixed effects regression modeling examining trajectory of concussive symptoms, mental health symptoms, sleep, health-related quality of life, and fear-avoidance during the Mobile Subthreshold Exercise Program (MSTEP) for concussion, Seattle, WA 2018–2019.

**Outcome**	**3 Weeks vs. Baseline**^****g****^		**6 Weeks vs. Baseline**^****g****^	
	***β* (95% CI)**	***P*-value**	***β* (95% CI)**	***P*-value**
HBI^a^	−7.12 (−12.50, −1.77)	0.01	−10.60 (−16.00, −5.10)	0.0002
GAD-7^b^	−2.61 (−4.78, −0.45)	0.019	−4.28 (−6.54, −2.02)	0.0003
PHQ-9^c^	−3.97 (−6.05, −1.89)	0.0002	−5.97 (−8.10, −3.85)	<0.0001
ASWS^d^	3.06 (−0.15, 6.27)	0.06	7.67 (4.40, 11.00)	<0.0001
PedsQL^e^	9.33 (2.85, 15.80)	0.005	15.10 (8.56, 21.60)	<0.0001
FOPQ^f^	−12.40 (−20.40, −4.42)	0.003	−21.60 (−29.80, −13.50)	<0.0001

a *HBI, Health and Behavior Inventory (concussive symptoms);*

b *GAD-7, Generalized Anxiety Disorder-7 item;*

c *PHQ9, Patient Health Questionnaire-9 item;*

d *ASWS, Adolescent Sleep Wake Scale;*

e *PedsQL, Pediatric Quality of Life Survey (youth self-report);*

f *FOPQ, Fear of Pain Questionnaire (youth self-report, adapted for concussive symptoms);*

g *All models controlled for child age, sex, duration of symptoms, and prior concussion history*.
